# Estimating proportions of missed children and reasons for non-vaccination following implementation of “reaching the unreached” multiantigen immunization campaign—south Khyber Pakhtunkhwa, Pakistan, July–August 2023

**DOI:** 10.3389/fpubh.2025.1591325

**Published:** 2025-09-29

**Authors:** Amen Ben Hamida, Fawad Alam, Brock Stewart, Shahid Ahmad, Eid Nawaz Sherani, Hafizullah Khan, Akerele Adekunle, Abdinoor Mohamed, Chukwuma Mbaeyi, Richard Franka, Abdul Basit, Rana Safdar

**Affiliations:** ^1^Global Immunization Division, U.S. Centers for Disease Control and Prevention (U.S. CDC), Atlanta, GA, United States; ^2^Integral Global Health, Islamabad, Pakistan; ^3^Khyber Pakhtunkhwa Province Polio Emergency Operations Centre, Peshawar, Pakistan

**Keywords:** polio, polio campaign monitoring, missed children, Pakistan, supplementary immunisation activities

## Abstract

During January 2022–June 2023, Pakistan reported 21 Wild Polio Virus 1 (WPV1) cases, all of which occurred within districts in the south Khyber Pakhtunkhwa (KP) province. In May 2023, a special immunization campaign was conducted to reach all children under 5 years of age within 69 high-risk union councils (UCs) in six districts of south KP. The campaign comprised of three rounds, each lasting 8 days, that provided bivalent oral poliovirus vaccine (bOPV) as well as other vaccines using a site-to-site delivery strategy. Rounds 1, 2, and 3 were conducted in July 2023, August 2023, and April 2024, respectively. We conducted a post-campaign evaluation (PCE) survey following the first two rounds, to assess OPV receipt, using a multistage sampling design. We analyzed PCE data for the two first rounds to provide UC-level estimates of the proportions of children who did not receive bOPV and assessed reasons for non-vaccination. The PCE survey included 8,125 children from 67 UCs during round 1 and 7,726 children from 47 UCs during round 2. The median number of villages by UC was 8 for both rounds. The median number of children by village was 16 for round 1 and 19 for round 2. Overall, 16% of children missed bOPV (95% CI = 14–18%) for round 1 [estimated total of 39,983 children (95% CI = 34,775–45,808)]; and 15% (95% CI = 13–17%) for round 2 [estimated total of 24,257 children (95% CI = 21,355–27,474)]. Percentages and numbers of missed children varied widely among UCs during both rounds. Six UCs in the first round and four UCs in the second had ≥40% missed children. Reasons for non-vaccination were similar for each round, with operational reasons leading by >60%, followed by refusals (≥20%), and child not available (~10%). We found a high proportion of missed children during this special immunization intervention and identified the UCs with the greatest challenges. In these UCs, there is a need to design and implement comprehensive, tailored, and effective interventions for each reason why children missed vaccination.

## Background

Poliomyelitis, commonly known as polio, remains a significant public health challenge, particularly in Pakistan and Afghanistan, the remaining two countries where indigenous wild poliovirus type 1 (WPV1) transmission persists (1). Despite substantial global progress toward bringing polio to the brink of eradication, these countries, which experience frequent cross-border population movement facilitating shared transmission, face unique challenges in eliminating transmission. In Pakistan, the persistence of poliovirus transmission results from several challenges, including population movements and density, inaccessibility of some areas due to security concerns, operational issues and vaccine resistance/hesitancy (2).

Approximately 20,000 wild poliovirus cases were reported annually in Pakistan in the early 1990s; however, by the early 2000s, the polio program has reduced this number by >99% (3). During January 2022–June 2023, Pakistan reported 21 WPV1 cases; all of these occurred within the southern districts of Khyber Pakhtunkhwa (KP) province, an area with considerable security challenges and a history of vaccine resistance (1). Pakistan has achieved this progress through strengthening of poliovirus surveillance and implementing multiple national and subnational supplementary immunization campaigns (SIAs), as well as enhancing essential (routine) childhood immunization (EI) activities (3).

Notwithstanding this progress, the ongoing transmission of WPV1 in Pakistan underscores the need for innovative strategies to vaccinate children who have consistently missed receipt of poliovirus vaccine (“missed children”) in SIAs and EI. The Global Polio Eradication Initiative (GPEI) has long recognized the need to address the “last mile” in polio eradication, which involves reaching the most vulnerable and subpopulations that are difficult to access (4). While an intensive schedule of SIAs and EI program strengthening have substantially reduced the burden of polio in Pakistan, a critical challenge remains in vaccinating children who are repeatedly missed by these vaccination activities. Populations unvaccinated with poliovirus vaccines can sustain ongoing WPV1 transmission (5). Addressing this transmission challenge requires innovative strategies to identify and vaccinate repetitively missed children, ensuring that no child is left behind.

In this context, the Pakistan Polio program in collaboration with Federal Directorate of Immunization devised a “special” immunization campaign called “reaching the unreached” (RUR) in May 2023 to be led by the provincial Extended Program on Immunization (EPI) of KP. The goal was to reach all children <5 years of age within 69 union councils (UCs) that were designated by the KP Emergency Operations Center (EOC) as “super high-risk,” through three rounds of immunization campaigns, providing bivalent oral poliovirus vaccine (bOPV) and other EPI vaccines. This initiative was also endorsed by the GPEI’s Technical Advisory Group on Poliomyelitis Eradication in Afghanistan and Pakistan in its meeting in June 2023 (6).

The RUR campaign, as a novel strategy within Pakistan’s polio eradication efforts, held the potential to provide valuable insights into the challenges of reaching zero-dose children. By evaluating this campaign, we aimed to quantify the extent to which children were missed for vaccination at a UC level, pinpoint areas facing the greatest obstacles, and elucidate the underlying reasons for non-vaccination to devise mitigation options. These can inform ongoing eradication activities in Pakistan and could also contribute to the global knowledge base on covering the “last mile” in polio eradication.

## Methods

### Reaching the unreached (RUR) initiative

In April 2023, the KP provincial EOC conducted a polio eradication limitations analysis of south KP’s 270 UCs. UCs were categorized as low-, medium-, high- and super high-risk based on a combination of indicators related to access, proportion of “fake finger-marking” in SIAs (fingernail marking falsely applied to children who had not received vaccine), SIA monitoring data, level of community engagement, size of high-risk and mobile populations, EI coverage, and surveillance performance. Sixty-nine UCs were flagged as “super high-risk” and consequently included for a “special” vaccine-preventable diseases (VPDs) and polio campaign (RUR) led by the Pakistan EPI in collaboration with the Polio program.

The RUR campaign followed a site-to-site delivery strategy in which field workers conducted house-to-house mobilization to inform parents about the timing of vaccination activities and location of vaccination posts, and the need for parents to take children to the designated sites to receive vaccination. Three rounds of 8 days each were planned, during which vaccinators provided bOPV as well as BCG, pentavalent vaccine (diphtheria-tetanus-pertussis-hepatitis B-*H. influenzae* type b) doses 1–3, inactivated poliovirus vaccine (IPV), and measles-rubella (MR) vaccine. The doses provided in these rounds were recorded in immunization cards used for tracking receipt of EI vaccines.

RUR rounds 1 and 2 (RUR1 and RUR2) were planned and conducted in July 2023 and August 2023. RUR round 3 (RUR3) was originally planned for September 2023 but postponed to April 2024 due to competing priorities, including the deployment of a rapid response team to investigate ongoing challenges in south KP and a national SIA implemented in late 2023.

### Post-campaign evaluation

Post-campaign evaluations (PCEs) were planned to follow all three RUR rounds. However, post-campaign evaluation for RUR3 was interrupted at its beginning due to the killing of a policeman who was accompanying the evaluators, raising the security risk. No RUR3 PCE data are included in this analysis.

According to the standard operating procedures, PCE is a rapid survey method used by the Pakistan Polio program to assess quality of vaccination activities in certain administrative areas such as UCs that are selected based on pre-defined criteria (e.g., all high-risk for polio, failed in previous polio post-campaign evaluations). Eight villages (“clusters” in our survey analyses) are selected from each UC following a simple random selection, from the village list that the polio program is maintaining. Ten households are selected among those having at least one child under 2 years of age by simple random selection. If a household does not respond, surveyors would move to the next household. All children <5 years of age in the HH who are present at the time of the visit are included in the survey. For each eligible child, surveyors asked the caretaker demographic data and assessed whether he/she had received bOPV in the most recent campaign (RUR1 or RUR2), by reviewing vaccination cards given during the campaign and by caretaker recall. For those children who had not received bOPV during the campaign, the surveyors asked for reasons for non-vaccination (single choice). Information on other vaccines was also collected, but it is outside the scope of the current analysis. Data collectors were trained on the use of the data collection app and the sampling methods.

The evaluators were solely responsible for conducting the survey, and did not vaccinate children who had missed doses during the rounds.

### Data source

Three Microsoft Excel datasets were used for this analysis: RUR1 and RUR2 PCE results for bOPV doses received, and the national polio program estimated target number of children under 5 years of age by UC for 2023.

### Reasons for non-vaccination

Reasons for non-vaccination with bOPV were categorized as follows: (1) Operational: includes “Family expected a house-visit by vaccination team,” “No vaccination activity in the area,” “Parents did not know about place, date or time of vaccination activity,” “Parents did not know about vaccination activities,” “Inconvenient vaccination site or time,” “Parents visited EPI center but vaccine was not given by vaccinator” and “Child was sick”; (2) Child not available: includes “Child was away from home and wasn’t available to participate in fixed-site vaccination activity”; (3) Refusals: includes “Fear of injection and adverse events following immunization, “Misconceptions” (e.g., vaccines cause disease, infertility, is not haram or is foreign agenda), “Parents refused polio vaccination of their child,” “Male members were not at home,” “Parents did not know the importance of vaccination,” and “Parents were busy”; and (4) Other.

### Data analysis

Only surveys with valid answers to the question about bOPV receipt during the rounds (Yes, No) were included in the analysis (one and two entries with no data for RUR1 and RUR2 PCE, respectively, were excluded). In addition, only UCs with more than one village selected for post-campaign evaluation were included in the estimates of missed (4 UCs were removed from RUR1 and 1 from RUR2). UCs were anonymized for the purpose of this publication while we identified the districts to which they belong.

Using the “survey” package of RStudio statistical software (7), we analyzed PCE data and provided UC-level estimates with 95% confidence intervals (CIs) of children who did not receive bOPV (missed children), accounting for multistage cluster survey design (8). Using targeted number of children, we estimated numbers of missed children by UC, with 95% CIs, using the “xlogit” method in R. For the UCs that had a post-campaign evaluation for both RUR1 and RUR2, we used the “svyglm” R function to run a logistic regression and compare the odds of missing children in both RUR1 and RUR2. Statistical significance was set at *p* = 0.05. Lastly, we analyzed reasons for non-vaccination by UC and campaign round.

This activity was reviewed by U.S. CDC, deemed not research, and was conducted consistent with applicable U.S. federal law and U.S. CDC policy.

## Results

Among the 69 UCs where two rounds of RUR were implemented, PCE activities were conducted in 67 UCs for RUR1 and 47 for RUR2 (Table 1). The median number of selected villages by UC was 8 for both rounds with a range of 1–9 for RUR1 and 1–8 for RUR2. The total number of selected villages was 444 for RUR1 and 361 for RUR2. The median number of children by UC was 121 for RUR1 (range = 22–283) and 173 for RUR2 (range = 18–316), for a total of 8,125 children for RUR1 and 7,726 for RUR2. The proportion of children under two was 64 and 86% in RUR1 and RUR2 PCEs, respectively.

**Table 1:** Numbers of Union Councils, Villages and Children included in the bOPV Post-Campaign Evaluation (PCE), Following “Reaching the Unreached” (RUR) Multi-Antigen, Fixed-Post Campaigns, by RUR Round—South Khyber Pakhtunkhwa, Pakistan, July–August 2023.

Sampling characteristics	RUR 1 PCE July 2023	RUR 2 PCE August 2023
Union Councils (UCs)	67	47
Median number of selected villages by UC (range)	8 (1–9)	8 (1–8)
Total villages selected	444	361
Median number of selected children by village (range)	16 (9–71)	19 (9–61)
Median number of selected Children by UC (range)	121 (22–283)	173 (80–316)
Total children selected	8,125	7,726

Among the 64 UCs that had a PCE with more than one cluster (village), 45 had a PCE in both, RUR1 and RUR2; 18 had a PCE only in RUR1; and 1 had a PCE only in RUR2. Overall, there were 16% missed children (95% CI = 14–18%) for RUR1 [corresponding to 39,983 children (95% CI = 34,775–45,808)]; and 15% missed children (95% CI = 13–17%) for RUR2 [corresponding to 24,257 children (95% CI = 21,355–27,474)].

**Figure 1:**
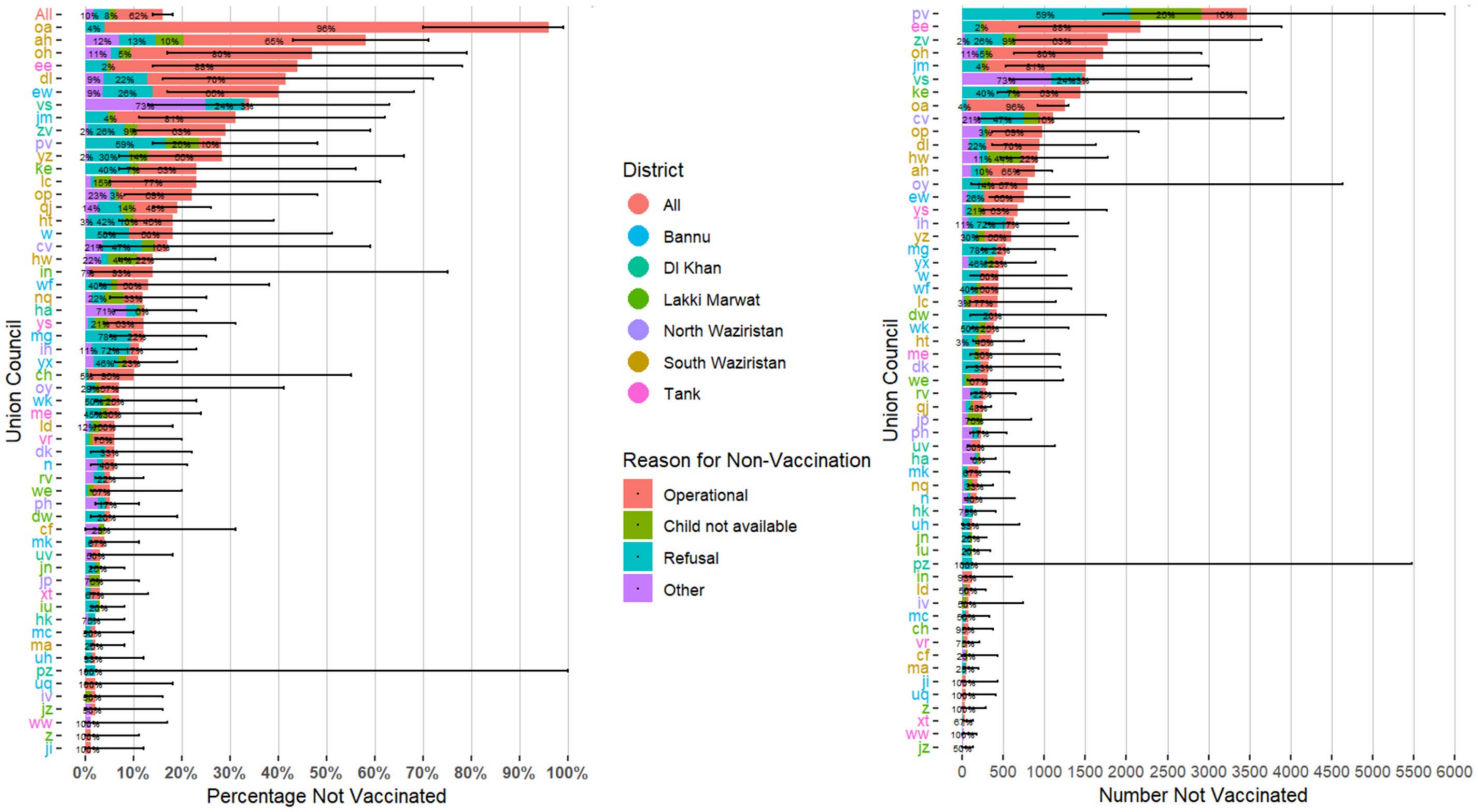
Estimated percentage and number of children who missed receipt of bOPV with 95% confidence intervals, by Union Council with stated reasons for non-vaccination (*N* = 57*)—Round 1 of Special “Reaching the Unreached” (RUR) Mult-Antigen, Fixed-Post Campaign, South Khyber Pakhtunkhwa, Pakistan, July 2023. *Union councils with only one village selected (*n* = 4) or with 0% missed children (*n* = 6) were not included in this figure. bOPV, bivalent oral poliovirus vaccine. Union councils’ names were anonymized for the purpose of this publication.

**Figure 2:**
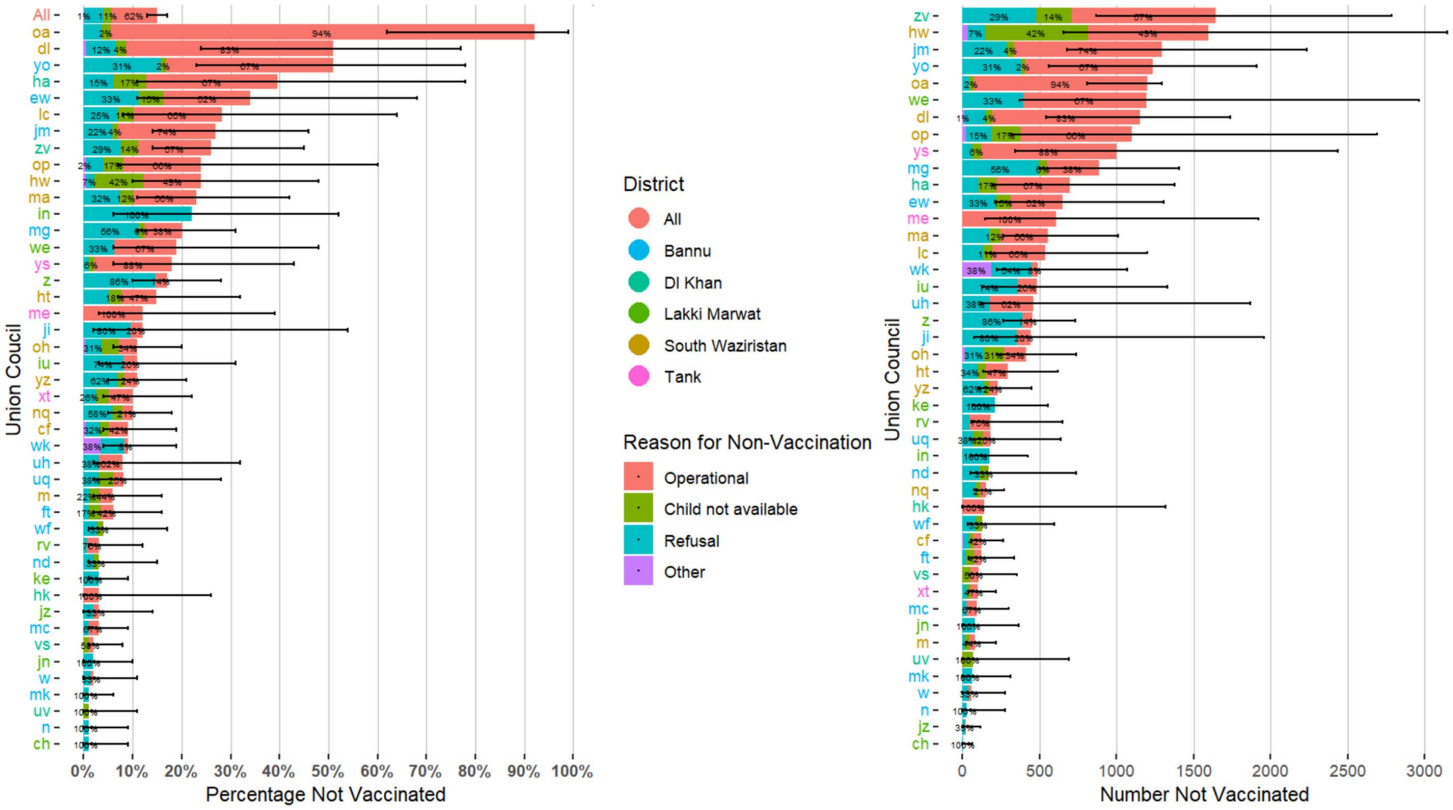
Estimated percentage and number of children who missed receipt of bOPV with 95% confidence intervals, by Union Council with stated reasons for non-vaccination (*N* = 44*)—Round 2 of Special “Reaching the Unreached” (RUR) Multi-Antigen, Fixed-Post Campaign, South Khyber Pakhtunkhwa, Pakistan, August 2023. *Union councils with only one village selected (*n* = 1) or with 0% missed children (*n* = 2) were not included in this figure. bOPV, bivalent oral poliovirus vaccine. Union councils’ names were anonymized for the purpose of this publication.

Percentages of missed children varied widely among UCs, ranging from 0 to 96% for RUR1 and from 0 to 92% for RUR2. There were 6 and 4 UCs that had 40% or more missed children during RUR1 and RUR2, respectively (Figures 1, 2). Estimates of the number of missed children ranged from 0 to 3,468 for RUR1 and from 0 to 1,641 for RUR2. There were 9 UCs during RUR1 and 8 during RUR2 that had 1,000 or more estimated missed children (Figures 1, 2).

**Figure 3:**
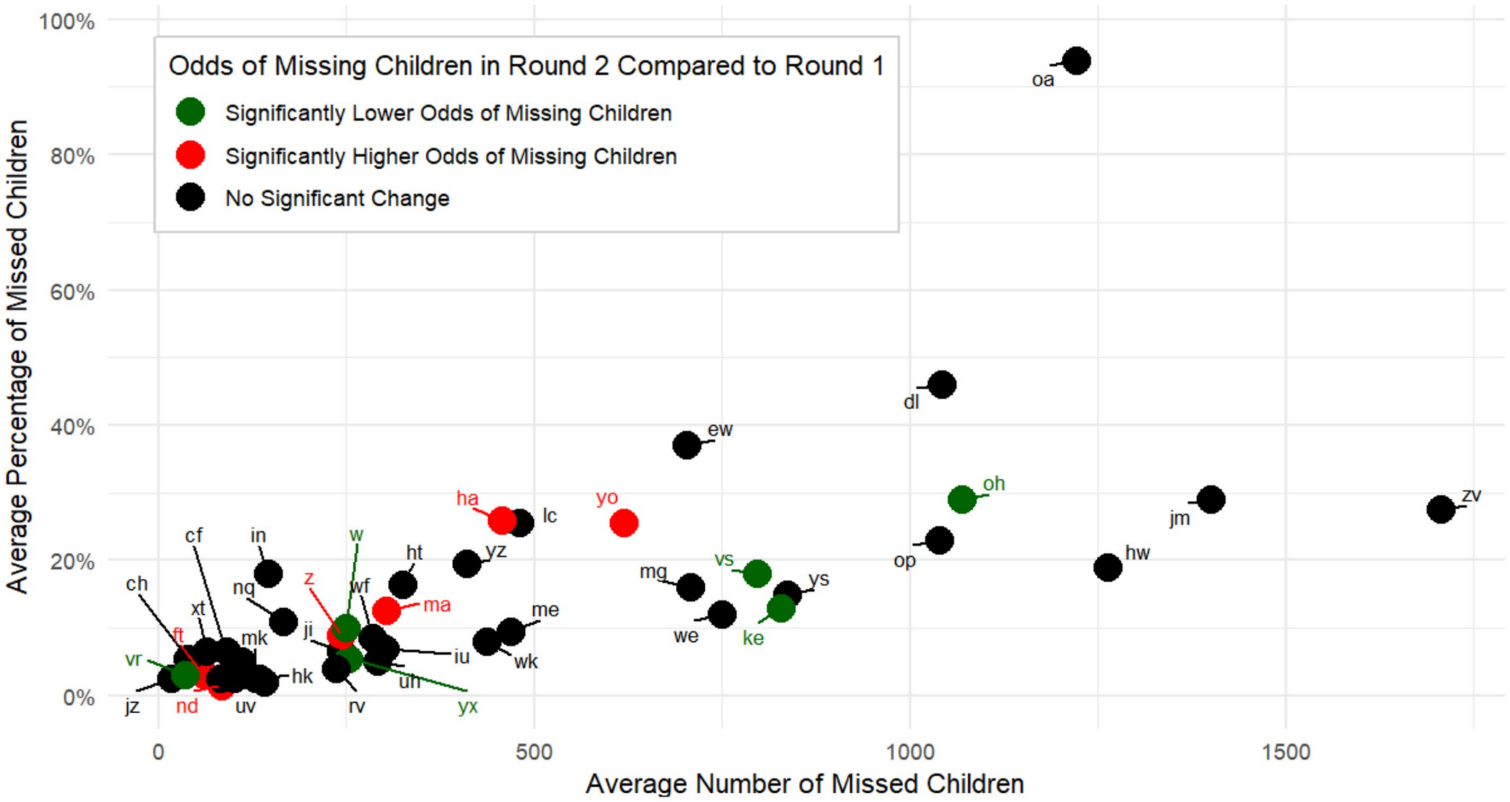
Comparison of average percentages and average numbers of children who missed receipt of bOPV during one or both rounds of “Reaching the Unreached” (RUR) Mult-Antigen, Fixed-Post Campaign, by Union Councils Evaluated Following Both RUR Rounds (*N* = 45)—South Khyber Pakhtunkhwa, Pakistan, July–August 2023. Statistical significance was defined as having a *p* value <0.05, using a logistic regression to compare the odds of missing children in the two rounds, and accounting for the complex survey design. UCs’ names were anonymized for the purpose of this publication. bOPV, bivalent oral poliovirus vaccine.

Among the 45 UCs that had a PCE conducted after both RUR1 and RUR2, 2 UCs had an average percentage of missed children >40% and an average number of missed children >1,000 (Figure 3). Six UCs had a statistically significant improvement in term of the odds of missing children between the two rounds, and 6 UCs had a statistically significant decline (Figure 3).

In term of reasons for non-vaccination, similar trends were observed in RUR1 and RUR2. For RUR1, 62% of reasons were “operational,” 20% were “refusals,” 8% were “child not available” and 10% were “other. For RUR2, 62% were “operational,” 26% were “refusals,” 11% were “child not available” and 1% were “other” (Figures 1, 2). “Operational” reasons for non-vaccination constituted ≥50% of total reasons for 31 and 19 UCs in RUR1 and RUR2, respectively (Figures 1, 2).

## Discussion

We estimated the proportion and number of missed children and reasons for non-vaccination with bOPV in 69 high-risk UCs, following a special integrated bOPV/multiantigen campaign, in 6 districts of South KP, Pakistan. Overall, percentages of missed children were similar between the two rounds [16% for RUR1 (95% CI = 14–18%), and 15% for RUR2 (95% CI = 13–17%)]. Reasons for non-vaccination also had similar trends between the two rounds, with “operational” reasons leading by more than 60%, followed by “refusals” (≥20%), “child not available” (~10%) and others. Although there was significant heterogenicity in the number of villages and children per village selected for the evaluation and wide confidence intervals in the estimates of missed children by UC, this analysis allowed the identification of UCs with the most significant challenges, including one UC that had >90% missed children in both rounds and 4 UCs that had ≥40% missed children in at least one round.

These UCs with high number of missed children in vaccination rounds pose a special risk to Pakistan’s polio eradication efforts, as reaching every child with bOPV is a necessary condition to achieve eradication goals (9). A recent analysis of program data found approximately 50,000 children to be regularly missed during OPV campaigns in the whole of south Khyber Pakhtunkhwa (1). Our findings estimated 39,983 missed children (95% CI = 34,775–45,808) for the 63 super-high-risk UCs of RUR1, and 24,257 missed children (95% CI = 21,355–27,474) for the 46 UCs of RUR2 included in this evaluation. Given that the UCs were not selected based on a random sampling, we cannot generalize findings to the whole of south Khyber Pakhtunkhwa. Notwithstanding the above, we have reasons to assume that the UCs excluded from this assessment would be missing less children in polio SIAs, than the UCs that were included, as the UCs selected for the RUR were considered to be very high risk based on a risk assessment, which included vaccination coverage, previous SIAs performance, population movements, and accessibility challenges. Furthermore, lot quality assurance sampling surveys, which assess SIA quality, continue to indicate substantial quality gaps in districts of south Khyber Pakhtunkhwa. Based on the 90% pass threshold, the proportion of UCs in south Khyber Pakhtunkhwa that reached this threshold ranged 56–80% for SIAs conducted during August 2022–February 2023 (1). Similarly, a cross-sectional survey conducted in 39 “super high-risk” UCs in three provinces (Sindh, Khyber Pakhtunkhwa and Baluchistan) showed that while 60.9% of children from these districts were vaccinated with at least three doses of OPV and one dose of IPV, 20.4% were under-vaccinated but with at least one dose OPV or IPV and 18.7% of children did not receive any polio vaccines (10).

Operational reasons were recorded as the main reasons for non-vaccination at UC level during the RUR in south KP (≥60%). Operational reasons are in theory within the control of the PEI and EPI programs and could represent an opportunity to assess and take corrective actions, especially needed in these UCs with the greatest challenges. However, it is also important to recognize that these areas are also among those with high insecurity, other access challenges, and high proportions of mobile populations (1, 2, 11, 12). For instance, the program was not able to access several areas among at least 14 UCs, due to security or community boycott reasons (around 37,500 unreached children). It is also worth noting that the post-campaign evaluation for RUR3 was interrupted due to a security incident. Close coordination with community members and leaders is essential to overcome these challenges.

Refusals were the second most common reason for non-vaccination in the two rounds (≥20%). Refusals are more challenging to address as they are multifactorial involving level of education of the parents, campaign “fatigue,” and cultural aspects (13, 14). A cross-sectional survey conducted within Quetta and Peshawar divisions in Pakistan showed that a large proportion of participants displayed negative attitudes towards polio immunization (85%), with lack of education and rural residence being significantly associated with the negative attitudes. Religious beliefs (39%), lack of knowledge about polio immunization (34%), fear of infertility by polio vaccines (32%) and security issues (29%) were reported by the participants as the main barriers towards polio immunization (13). Another survey among parents of persistently missed children in the high-risk areas of Karachi found that among refusals, 37% had no trust in vaccine quality, 15% were afraid of side effects, 14% were not allowed by their elders, 13% refused due to the influence of negative social media videos, and 6% had no trust in polio teams (non-exhaustive list of reasons) (15). These challenges need to be addressed by the program through educational media and community outreach campaigns involving health professionals, community leaders and religious influencers (15).

This assessment has several limitations. First, not all UCs had a post-campaign evaluation in both rounds, and UCs with only one village selected were removed from analysis due to the statistical technique used to assess CIs. Results of this analysis are only representative of the UCs that were included in the special campaigns and assessment (i.e., not representative of all 270 UCs of south Khyber Pakhtunkhwa). Additionally, villages were sampled randomly from the UCs. Accurate and complete data on villages sampling and sizes was not available to the team to conduct adequate weighting. Thus, UC estimates could be slightly skewed due to differences in villages size. Second, only children that were present in the households at the time of visit were included, which may overestimate vaccination coverage if the same children who were missed by vaccinators were also missed by surveyors. Third, the surveyors only visited a small number of clusters and due to security limitations, were not always able to reach the desired target of 8 clusters (47 and 15% of UCs had less than 8 clusters in RUR1 and RUR2, respectively), which resulted in large CIs for estimates of children missed and included potential bias. However, we believe the point estimates and these CIs are still useful to guide the program, especially for the lowest performing UCs, where the lower end of the CI is still too high for eradication goals. Fourth, updated census data by age category were not available at the time of the analysis. The polio program’s number of targeted children by UC were consequently used as estimates of the population <5 years of age by UC, which could be misestimates of the true population numbers. Lastly, due to the multiantigen nature of the RUR campaign, the government of Pakistan opted for a site-to-site strategy for this campaign, which tends to be less effective compared to the house-to-house strategy (vaccinators visit and vaccinate eligible children in all houses in an area), in reaching all targeted children for polio-specific immunization campaigns (16, 17).

Despite these limitations, we believe this analysis provides critical information to identify UCs with the most challenges and guide targeted support to these communities with most significant needs. Moving forward, there is a need for the Pakistan Program to standardize post-campaign evaluation sampling methods, especially in relation to the number of villages per UC and households per village, and to advocate for a higher number of villages and households recruited in order to have more accurate, precise, and generalizable estimates. There is also a need to further explore the available data and eventually collect new information to understand the specific challenges of outlying UCs (e.g., with very high percentages of missed children, operational reasons, or other) in order to design interventions that are tailored to their specific needs.

Considering the multiple challenges to vaccinating children in south Khyber Pakhtunkhwa, the EPI and polio programs should continue to collaborate on innovative strategies in order to reach children and address all operational challenges to vaccination.

## Data Availability

The datasets presented in this article are not readily available because data owned by the Polio Pakistan program. Requests to access the datasets should be directed to RS, ranasafdar@integralglobal.net.

## References

[1] Mbaeyi C. Progress toward poliomyelitis eradication—Pakistan, January 2022–June 2023. MMWR Morb Mortal Wkly Rep. (2023) 72:880–5. doi: 10.15585/mmwr.mm7233a1, PMID: 37590173 PMC10441828

[2] Hussain SF Boyle P Patel P Sullivan R. Eradicating polio in Pakistan: an analysis of the challenges and solutions to this security and health issue. Glob Health. (2016) 12:1–9. doi: 10.1186/s12992-016-0195-3, PMID: 27729081 PMC5059991

[3] Ataullahjan A Ahsan H Soofi S Habib MA Bhutta ZA. Eradicating polio in Pakistan: a systematic review of programs and policies. Expert Rev Vaccines. (2021) 20:661–78. doi: 10.1080/14760584.2021.1915139, PMID: 33896306

[4] GPEI (2022) Polio eradication strategy 2022-2026: delivering on a promise. Available online at: https://polioeradication.org/wp-content/uploads/2022/06/Polio-Eradication-Strategy-2022-2026-Delivering-on-a-Promise.pdf (Accessed September 16, 2025).

[5] Hogan D Gupta A. Why reaching zero-dose children holds the key to achieving the sustainable development goals. Vaccine. (2023) 11:781. doi: 10.3390/vaccines11040781, PMID: 37112693 PMC10142906

[6] GPEI. (2023). Meeting report of the technical advisory group on poliomyelitis eradication in Afghanistan and Pakistan. Available online at: https://polioeradication.org/wp-content/uploads/2023/09/Meeting-Report-of-the-Technical-Advisory-Group-on-Polio-Eradication-in-Afghanistan-and-Pakistan-June-2023.pdf (Accessed September 13, 2024).

[7] The R Foundation (2024) The R project for statistical computing. Available online at: https://www.r-project.org/

[8] Thomas Lumley P. G. Schneider Ben. (2024). Survey: analysis of complex survey samples. Available online at: https://cran.r-project.org/web/packages/survey/index.html

[9] Zaffran M McGovern M Hossaini R Martin R Wenger J. The polio endgame: securing a world free of all polioviruses. Lancet. (2018) 391:11–3. doi: 10.1016/S0140-6736(17)32442-X, PMID: 29323639

[10] Khan A Hussain I Rhoda DA Umer M Ansari U Ahmed I. Determinants of immunization in polio super high-risk union councils of Pakistan. Vaccine. (2024) 42:583–90. doi: 10.1016/j.vaccine.2023.12.056, PMID: 38143197 PMC10850981

[11] Molodecky NA Usman A Javaid A Wahdan A Parker EP Ahmed JA. Quantifying movement patterns and vaccination status of high risk mobile populations in Pakistan and Afghanistan to inform poliovirus risk and vaccination strategy. Vaccine. (2021) 39:2124–32. doi: 10.1016/j.vaccine.2021.03.001, PMID: 33736917

[12] Verma AA Jimenez MP Tangermann RH Subramanian S Razak F. Insecurity, polio vaccination rates, and polio incidence in Northwest Pakistan. Proc Natl Acad Sci USA. (2018) 115:1593–8. doi: 10.1073/pnas.1711923115, PMID: 29378938 PMC5816148

[13] Khan MU Ahmad A Aqeel T Salman S Ibrahim Q Idrees J. Knowledge, attitudes and perceptions towards polio immunization among residents of two highly affected regions of Pakistan. BMC Public Health. (2015) 15:1–8. doi: 10.1186/s12889-015-2471-1, PMID: 26541976 PMC4635542

[14] Saeed H Azhar S Syed A Khalid S Bukhari A Saeed N. Polio and its vaccination: a cross sectional study of knowledge, attitude and perception of general public in district Abbottabad and Mansehra, Khyber Pakhtunkhwa, Pakistan. Anti-Infect Agents. (2018) 16:22–31. doi: 10.2174/2211352516666180126161803

[15] Abbasi FH Shaikh AA Mehraj J Raza SM Rasool S Bullo UF. Vaccine hesitancy and perceptions of the community about polio in high-risk areas of Karachi, Sindh, Pakistan. Vaccine. (2022) 11:70. doi: 10.3390/vaccines11010070, PMID: 36679915 PMC9866813

[16] Curry DW Perry HB Tirmizi SN Goldstein AL Lynch MC. Assessing the effectiveness of house-to-house visits on routine oral polio immunization completion and tracking of defaulters. J Health Popul Nutr. (2014) 32:356. https://pmc.ncbi.nlm.nih.gov/articles/PMC4216971/ (Accessed September 16, 2025). 25076672 PMC4216971

[17] Linkins R Mansour E Wassif O Hassan M Patriarca PA. Evaluation of house-to-house versus fixed-site oral poliovirus vaccine delivery strategies in a mass immunization campaign in Egypt. Bull World Health Organ. (1995) 73:589. https://pubmed.ncbi.nlm.nih.gov/8846484/ (Accessed September 16 2025). 8846484 PMC2486824

